# PReS-FINAL-2279: Lupus nephritis in a Colombian cohort of pediatric patients

**DOI:** 10.1186/1546-0096-11-S2-P269

**Published:** 2013-12-05

**Authors:** AS Diaz Maldonado, F Gonzalez, A Monje Gaitan, N Gamba

**Affiliations:** 1 Reumatologia Pediatrica, Hospital de La Misericordia, Bogota, Colombia

## Introduction

Renal involvement is a major cause of morbidity and hospital admissions in systemic lupus erythematosus (SLE) patients and occurs in 40% to 70% of all patients. Generally, renal involvement tends to occur within the first 2 years of SLE with its frequency decreasing significantly after the first 5 years of disease.

## Objectives

To describe and to compare the clinical features and disease activity of a cohort of patients with SLE at a Colombian children's hospital in two different moments.

## Methods

Analytic descriptive study. 89 patients with diagnosis of SLE (1997 ACR revised criteria) from a rheumatology center at a pediatric hospital were evaluated at the time of diagnosis and twelve months after. Medical records were reviewed registering the following variables: Sex, age, renal involvement^1 ^(Yes/No), hypertension (Yes/No), nephrotic range proteinuria^1 ^(Yes/No), renal biopsy (Yes/No), histological classification of lupus nephritis (HCLN) (International Society of Nephrology/Renal Pathology Society, 2003), dialysis (Yes/No), and score Systemic Lupus Erythematosus Disease Activity Index (SLEDAI). Analysis was done through parametric and non parametric tests to compare means and proportions, using STATA11.

## Results

76 patients (85.4%) were female and 13 (14.6%) were men. Mean age at the time of diagnosis was 11,3 years old (Min 2, Max 16). Renal involvement at the time of diagnosis was found in 73 patients (82%); after 12 months it was found in 68 patients (76,4%) (p < 0,01), hypertension at the time of diagnosis was found in 30 patients (41,1%); after 12 months it was found in 8 patients (11%) (p < 0,01), nephrotic range proteinuria at the time of diagnosis was found in 15 patients (20,5%); after 12 months it was found in 8 patients (11%) (p < 0,01), 36 patients (49,3%) underwent renal biopsy, 16 patients (44,4%) underwent a second renal biopsy, HCLN first biopsy: Class I (8,3%), Class II (11,1%), Class III (8,3%), Class IV (50%), Class V (13,9%), Class III-IV (5,6%), HCLN second biopsy: Class I (6,3%), Class III (6,3%), Class IV (62,5%), Class V (25%), Class IV-V (12,5%), patients on dialysis at the time of diagnosis were 1 (1,4%); after 12 months they were 3 (4,4%) (p = 0,36), and median score SLEDAI at the time of diagnosis was 23 (Min 4, Max 61); after 12 months it was 4 (Min 0, Max 31) (p < 0,01).

## Conclusion

The most common HCLN in this cohort were Class IV, and Class V. Statistical significance (difference) was documented regarding renal involvement, hypertension, nephrotic range proteinuria, and median score SLEDAI for the time of diagnosis and twelve months follow-up. This suggests that the change and improvement in clinical features and disease activity was due to the established treatment.

## Disclosure of interest

None declared.

